# Aquatic biosystems: reactions and actions

**DOI:** 10.1186/2046-9063-8-1

**Published:** 2012-01-30

**Authors:** Edward J Phlips, Shiladitya DasSarma

**Affiliations:** 1 Department of Fisheries and Aquatic Sciences, 7922 NW 71st Street, University of Florida, Gainsville, FL 32653, USA; 2 Department of Microbiology and Immunology, Institute of Marine and Environmental Technology, 701 East Pratt Street, University of Maryland, Baltimore, MD 21202, USA

## Abstract

Aquatic biological systems are a critical part of the structure and function of earth's biosphere. While attention of the scientific community is often focused on the reaction of biological systems to changes in the environment, these systems also have profound effects, or actions, on the environment. Throughout the evolutionary history of earth, the rise and/or fall of different aquatic biosystems has impacted the character of the biosphere. At no time have environmental changes been more important to all life on earth than in the modern era, which underscores the need for the new journal, *Aquatic Biosystems*. We welcome submission of original research manuscripts, reviews, and commentaries to the journal.

## Commentary

While the nature of the last common ancestor of all life remains enigmatic, geological evidence indicates the evolution of diverse prokaryotic microorganisms early in the earth's history. Around 3 billion years ago, the concerted metabolic activities of oxygen-evolving aquatic photosynthetic bacteria led to an oxygen-enriched atmosphere, and the development of a UV-protective ozone blanket, both of which provided the antecedents for an explosion of biological diversity and biomass. Two billion years later, the rise of larger and structurally more complex algae and aquatic plants provided new habitats and energy sources for the expansion of aquatic animal populations, which in turn formed the basis of new food webs.

Subsequent shifts in the dominance structure of aquatic biological communities, driven in part by continental drift and sea level changes, influenced the chemical and geological character of the biosphere. A primary example of such a shift in dominance was the proliferation of planktonic coccolithiphores in the early cretaceous, leading to vast calcium carbonate deposits on the ocean floor and alterations in ocean chemistry. Modern studies of the ocean floor are leading to discoveries of complex communities, such as near hydrothermal vents and submarine brines, which may represent a snapshot of the early history of planet earth.

Most recently, during the past few ticks of the evolutionary clock, the rapid surge in human population and development has reached a scale and dimension that can significantly alter the structure and function of aquatic biosystems, even on a global scale. The increasing and widening threats posed by the actions of human biosystems to the integrity and sustainability of aquatic biosystems highlight the importance of understanding how these systems function, and their resilience to environmental change. Understanding the aquatic microbial community and its effects on plants and animals is key to choosing a sustainable future. It is also important to place the reactions of aquatic biosystems to human actions within the context of non-anthropogenically-driven changes in the environment, such as climatic cycles.

The complexity of this task will require the use of all available resources, including the wide range of technological capabilities driving basic and applied research in the 21^st ^century. Success will require open access to information and the concerted and long-term commitment of scientists, managers and policy makers. This commitment must extend to exploring innovative ways to prevent, minimize or reverse damage to aquatic biosystems, including the development of engineered biosystems aimed at renewable production of essential resources and mitigation of harmful waste materials, such as greenhouse gases, excess nutrients and pollutants. In other words, countering the consequences of negative actions with positive actions, in order to avoid negative reactions by critical aquatic biosystems.

In order to facilitate communication across such an interdisciplinary spectrum, we launch the new Open Access independent journal named *Aquatic Biosystems*. The scope of the journal ranges from the molecular and organismic levels to global systems and processes, reflecting the wide range of research in aquatic biological systems. The journal encourages reports on a wide array of environments, including lakes, rivers, marshes, springs, lagoons, solar pans, estuaries, and the open seas and ocean, and their micro- and macro-flora and fauna. The focus is on the relationships between the environment and biological systems, encompassing microbial genomics, physiology, and ecology, biogeochemical cycling, food webs, paleolimnology, biodiversity, conservation, resource management, and ecosystem structure and function. Engineered systems, such as for aquaculture, renewable resource, biofuels, biotechnology, and biomedical production, as well as constructed wetlands, are within the scope of *Aquatic Biosystems*.

The goal of *Aquatic Biosystems *is to bridge across freshwater and saline systems, between basic and applied research, and from gene systems to ecosystems. The online Open Access format of the journal is designed to accelerate the process of disseminating important research results and information, in order to better meet the needs and demands of the highly dynamic and global science, management, and private sectors. We invite our international community of fellow scientists to contribute to *Aquatic Biosystems*.

